# The efficacy of lactic acid bacteria-based toothpaste on oral health: a systematic review and meta-analysis

**DOI:** 10.3389/froh.2025.1668943

**Published:** 2025-10-20

**Authors:** Eun-Mi Choi, Su-Kyung Park

**Affiliations:** 1 Department of Dental Hygiene, Daejeon Health University, Daejeon-si, Republic of Korea; 2 Department of Dental Hygiene, Graduate School, Yonsei University, Wonju-si, Republic of Korea

**Keywords:** dental plaque, gingivitis, lactic acid bacteria, oral microbiome, periodontal disease, probiotics, toothpaste

## Abstract

**Introduction:**

Lactic acid bacteria (LAB) have emerged as promising adjunctive agents for oral health management due to their antimicrobial and immunomodulatory properties. With the increasing incorporation of probiotics into oral care products, it is critical to evaluate their clinical efficacy. This systematic review and meta-analysis aimed to assess the effectiveness of LAB-based toothpaste in improving oral health outcomes.

**Methods:**

Following PRISMA guidelines, five databases (MEDLINE, EMBASE, Scopus, Web of Science, and CENTRAL) were searched through February 2025. Randomized controlled trials (RCTs) evaluating toothpastes containing probiotic, prebiotic, synbiotic, or postbiotic agents were included. Primary outcomes included plaque index (PI), gingival index (GI), bleeding on probing (BOP), probing pocket depth (PPD), and clinical attachment level (CAL). Secondary outcomes assessed oral microbiota changes. Risk of bias was evaluated using the RoB 2 tool.

**Results:**

Twelve RCTs were included, with four studies suitable for meta-analysis. Meta-analysis demonstrated significant plaque reduction at 3 months [Mean Difference (MD) = −0.64; 95% confidence interval (CI): −1.14 to −0.15; *p* = 0.01] and BOP improvement (MD = −1.49; 95% CI: −2.42 to −0.56; *p* = 0.002). Longer interventions (≥6 months) in periodontitis patients revealed significant PPD reduction (MD = −1.32; 95% CI: −1.81 to −0.84; *p* < 0.00001) and CAL improvement (MD = −0.79; 95% CI: −1.25 to −0.33; *p* = 0.0007). Streptococcus mutans levels were significantly reduced across multiple studies.

**Conclusions:**

LAB-based toothpaste demonstrates beneficial effects on plaque control and gingival inflammation. However, substantial heterogeneity (I^2^ > 75% for most outcomes) limits effect estimate precision. Lactobacillus paracasei strains showed consistent benefits, while sustained use (≥6 months) appears necessary for periodontal improvements.

**Systematic Review Registration:**

https://www.crd.york.ac.uk/prospero/display_record.php?ID=CRD420250650340, PROSPERO CRD420250650340.

## 1 Introduction

Lactic acid bacteria (LAB) have garnered significant attention as adjunct therapeutic agents for maintaining oral health, particularly in managing periodontal diseases. The oral microbiome, a complex and dynamic ecosystem composed of bacteria, fungi, and viruses, plays a pivotal role in maintaining microbial homeostasis. Disruptions in this equilibrium, referred to as dysbiosis, contribute to the pathogenesis of oral diseases and have been associated with systemic conditions such as diabetes and cardiovascular disease (1). Beneficial bacteria such as *Rothia* and *Neisseria* species demonstrate potential for promoting oral health through pathogen inhibition and immune response modulation (2). Among various probiotic approaches for oral health, LAB demonstrates superior oral adaptation through enhanced persistence and targeted antimicrobial activity against oral pathogens (3). This makes LAB particularly suitable for oral health interventions.

Recent advances in microbiome research have facilitated numerous clinical investigations, with over 200 registered randomized controlled trials (RCTs) currently investigating probiotic applications for periodontitis, gingivitis, dental caries, and peri-implant diseases (4). These extensive research efforts have particularly focused on LAB due to their demonstrated clinical efficacy and safety profile in oral applications.

The mechanisms through which probiotic LAB contribute to oral health maintenance include: (i) competitive inhibition for adhesion sites and nutrients; (ii) antimicrobial agent production including bacteriocins; (iii) pathogenic microorganism growth and biofilm formation inhibition; (iv) direct pathogen interactions including coaggregation; (v) neutralization of pathogen-produced cytotoxic metabolites; and (vi) modulation of local and systemic immune responses (3).

A 2022 meta-analysis by Gheisary et al. demonstrated that probiotics can improve clinical parameters such as gingival index (GI), plaque index (PI), and bleeding on probing (BOP) in patients with periodontal disease by effectively inhibiting periodontal pathogens and pro-inflammatory factors in the oral cavity (5). Additionally, studies have shown that Lactobacillus reuteri-containing probiotic formulations can effectively improve periodontal clinical indices. Lactobacillus species may play a significant role in inhibiting dental plaque formation by competing for binding sites, thereby preventing colonization by pathogenic oral bacteria (6).

Furthermore, Chen et al. reported that heat-killed probiotic strains, including several Lactobacillus strains and *Streptococcus animalis* subsp. lactis AP-32, exhibited direct antibacterial activity against oral pathogens, such as *Streptococcus mutans, Porphyromonas gingivalis*, and *Fusobacterium nucleatum* (7).

The increasing recognition of the beneficial role of LAB in oral healthcare has facilitated their incorporation into various oral care products, including microbiome-based toothpastes designed to restore and maintain microbial balance. As interest in microbiome-based interventions continues to expand, conducting systematic reviews is crucial for developing effective strategies to optimize the use of LAB in oral care products to maintain oral microbial balance and prevent disease.

## 2 Methods

This systematic review was conducted in accordance with the Preferred Reporting Items for Systematic Reviews and Meta-Analyses (PRISMA) guidelines (8). The study protocol was registered in the PROSPERO International Prospective Register of Systematic Reviews (CRD420250650340).

### 2.1 Research question and eligibility criteria

The research question for this systematic review was formulated using the PICO (Patient, Intervention, Comparison, and Outcome) framework, leading to the following question: “What is the efficacy of LAB-based toothpaste (containing synbiotics, prebiotics, probiotics, or postbiotics) in improving oral health outcomes compared to placebo, non-pharmacological treatments, or no treatment?”

#### 2.1.1 Participants

Adults and children with healthy gingiva or periodontal disease were included. Adults and children (13–15 years) were combined as a single population because this pediatric age group represents adolescents with fully established permanent dentition, ensuring comparable target pathogenic bacteria and consistent methodological protocols across age groups. Exclusion criteria comprised: individuals undergoing active dental treatment; those who had used systemic antibiotics, anti-inflammatory drugs, or probiotic preparations within three months; and individuals with systemic diseases or conditions potentially interfering with study results (e.g., pregnancy, immunological disorders).

#### 2.1.2 Intervention

LAB-based toothpaste containing synbiotic, prebiotic, postbiotic, or probiotic agents.

#### 2.1.3 Comparison

Placebo, conventional toothpaste, or no treatment.

#### 2.1.4 Outcome

Primary outcomes included oral health parameters [PI, GI, BOP, PPD, clinical attachment level (CAL)]. Secondary outcomes comprised oral microbiota changes.

### 2.2 Criteria for considering studies for this review and search strategy

All RCTs and clinical trials were included. Single-arm studies, commentaries, editorials, systematic reviews, clinical observations, and articles without abstracts were excluded. We searched MEDLINE (via PubMed), EMBASE (via Ovid), Scopus, Web of Science, and the Cochrane Central Register of Controlled Trials (CENTRAL). The last search of all databases was conducted on February 15, 2025. Complete search strategies for all databases are provided in Supplementary Material 1. No restrictions were imposed on language or publication year. Additional resources searched included gray literature sources such as abstracts, dissertations, and theses.

#### 2.2.1 Ovid MEDLINE search strategy

(“probiotics”/exp OR “probiotics”:ab,ti OR “prebiotics”/exp OR “prebiotics”:ab,ti OR “synbiotics”/exp OR “synbiotics”:ab,ti OR “postbiotics”:ab,ti OR “bifidobacterium bifidum”/exp OR “*lactobacillus*”/exp OR “bacillus”/exp OR “probiotic*” OR “prebiotic*” OR ”synbiotic*” OR “postbiotic*” OR “bacillus*”) AND (“dentifrices”/exp OR “dentifrices”:ab,ti OR “dentifrice*” OR “toothpastes”/exp OR “toothpastes”:ab,ti OR “toothpaste*”) AND (“randomized controlled trial”:ab,ti OR “controlled clinical trial”:ab,ti OR “randomized”:ab,ti OR “placebo”:ab,ti OR “randomly”:ab,ti OR “trial”:ab,ti OR “groups”:ab,ti).

### 2.3 Screening and selection

**Figure 1:**
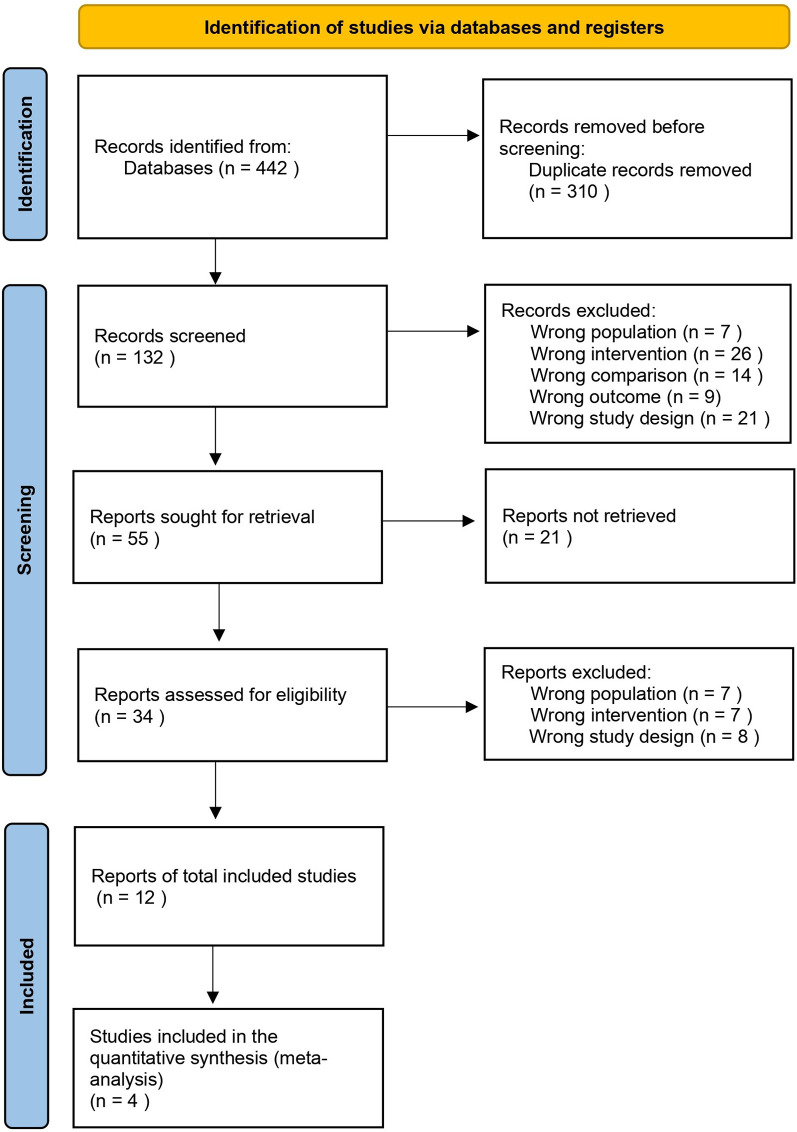
PRISMA flow diagram illustrating the study selection process.

All retrieved records were imported into EndNote reference management software for initial organization. Duplicate detection and removal were performed using the Rayyan web-based systematic review platform (9), with manual verification to ensure completeness. Two independent reviewers (EM and SK) then assessed titles and abstracts using the same platform, independently applying predetermined inclusion and exclusion criteria during the initial screening phase. Full-text assessment was performed independently by the same reviewers for all potentially eligible studies. Disagreements during title/abstract screening and full-text assessment were resolved through discussion between the two reviewers. All excluded full-text articles were documented with specific reasons for exclusion according to predetermined criteria. The complete study selection process is illustrated in Figure 1 using a PRISMA flow diagram.

### 2.4 Data extraction

Data extraction was performed independently by two authors (EM and SK), who systematically documented pertinent information using a standardized extraction form. The collected data included the first author, publication year, study aim, sample size, study design and duration, test and control regimens, demographic characteristics of participants, primary and secondary outcomes, and the original authors' conclusions.

### 2.5 Data analysis

The primary outcomes of interest were oral health-related parameters, including GI, PI, BOP, oral hygiene, and oral disease. Additionally, other parameters indicative of oral health were investigated, such as the periodontal pathogens *Lactobacillus*, Bifidobacterium, and Bacillus. Meta-analysis was performed using Review Manager 5.4 software. Meta-analysis was conducted according to PRISMA guidelines. Standard deviations (SDs) were estimated using confidence interval (CI) limits or standard errors. The precision of effect sizes was reported with a 95% CI. Pooled outcomes were expressed as weighted mean difference (MD), and statistical heterogeneity among studies was assessed using Cochran's Q statistic and I^2^. We interpreted I^2^ values according to the Cochrane Handbook guidelines: 0%–40% might not be important; 30%–60% may represent moderate heterogeneity; 50%–90% may represent substantial heterogeneity; and 75%–100% represents considerable heterogeneity. The importance of observed I^2^ values was evaluated considering the magnitude and direction of treatment effects and the strength of evidence for heterogeneity (*P*-value from the Chi^2^ test). Given the expected clinical and methodological diversity among studies, we used random-effects models for all meta-analyses. When substantial heterogeneity (I^2^ > 75%) was observed, we interpreted results with appropriate caution and explored potential sources of variation through examination of the study characteristics. Given the small number of studies available for several meta-analyses (ranging from 2 to 4 studies), we acknowledge that between-study variance estimates may be imprecise and confidence intervals should be interpreted with caution. When substantial heterogeneity was observed, results were interpreted with caution and sources of variation were explored through subgroup analyses (population and strain).

### 2.6 Risk of bias assessment

**Figure 2:**
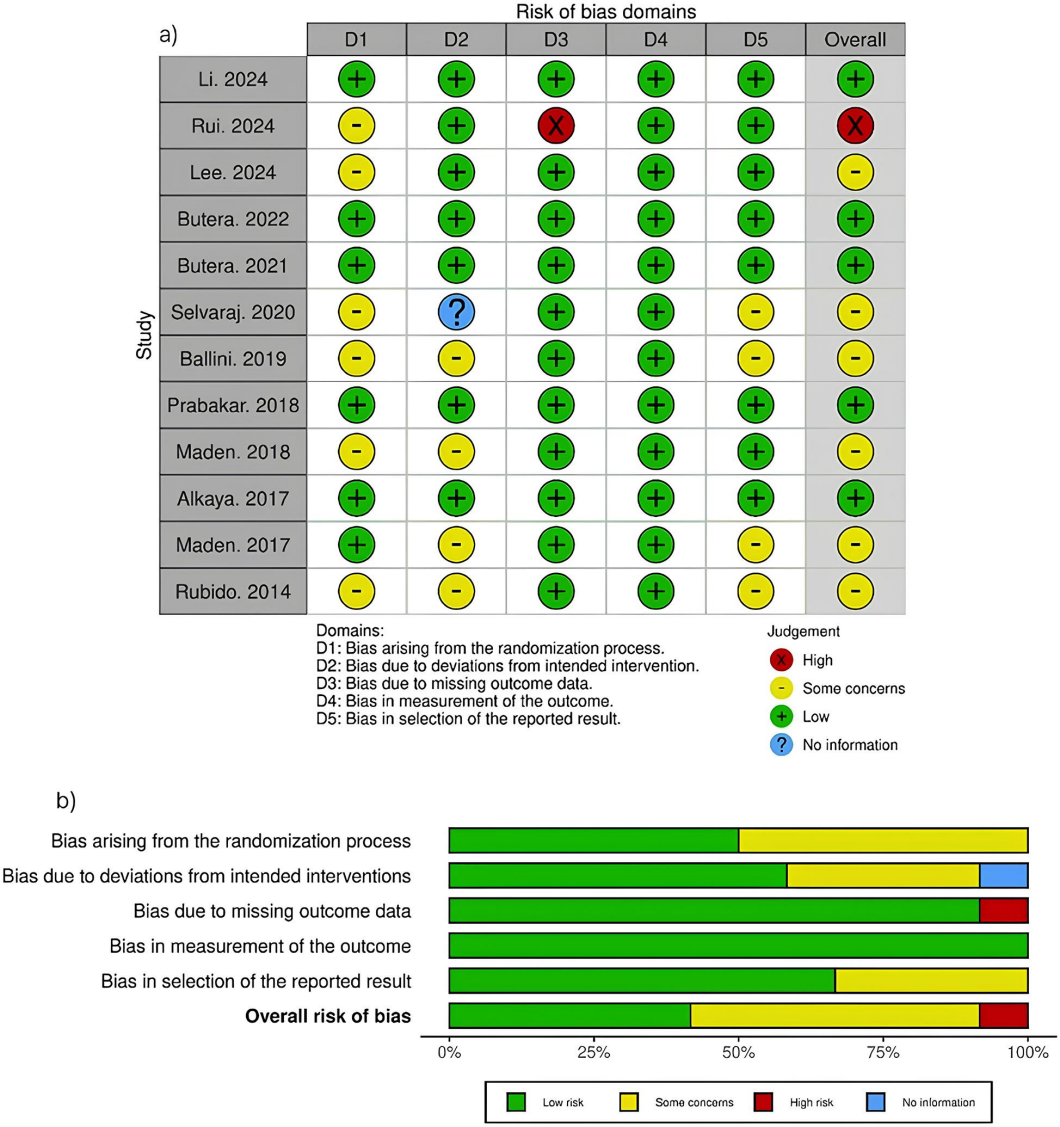
Risk of Bias assessment as traffic light plot **(a)** and weighted bar plot **(b)**.

Risk of bias for each included study was independently assessed by two authors (EM and SK) using Cochrane's Risk of Bias Version 2 (RoB 2) (10). This assessment examined five domains: the randomization process, deviations from intended interventions, missing outcome data, measurement of outcomes, and selection of reported results. Figure 2 presents a comprehensive visualization of these risk of bias domains. Any discrepancies between reviewers were resolved through collaborative discussion.

### 2.7 Quality of the evidence

We used the GRADE approach, as described in the Cochrane Handbook for Systematic Reviews of Interventions, to assess the quality of the body of evidence for the primary outcomes (11). The quality of a body of evidence for a specific outcome is based on five factors: (1) limitations of the study designs; (2) indirectness of evidence; (3) inconsistency of results; (4) imprecision of results; and (5) publication bias. The GRADE approach specifies four levels of quality (high, moderate, low, and very low), incorporating the factors noted above. Quality of evidence by GRADE should be interpreted as follows: High-quality: The likelihood that the effect will be substantially different is low.; Moderate-quality: The true effect is likely to be close to the estimate of the effect, but there is a possibility that it is substantially different Low-quality: The likelihood that it will be substantially different is high; Very low-quality: The true effect is likely to be substantially different from the estimate of effect.

## 3 Results

### 3.1 Study selection

The database search identified 442 records, with 132 remaining records after duplicate removal. Following title/abstract screening, 34 full-text articles were assessed for eligibility. Twelve studies met the inclusion criteria and were included in the final analysis (12–23).

### 3.2 Study characteristics

The included studies, published between 2014 and 2024, were all RCTs with sample sizes ranging from 17 to 92 participants. Study populations included patients with periodontitis (*n* = 2) (15, 16), patients with gingivitis (*n* = 3) (12, 14, 21), and healthy individuals (*n* = 8) (12, 13, 17–20, 22, 23). Treatment durations ranged from 1 day to 6 months, with most implementing twice-daily protocols over 2–6 months (Table 1).

**Table 1:** Characteristics of the included studies.

No.	Year	Country	Population (Age)	Intervention type and dose	Comparison	Duration	Outcome measures	Conclusion
1	Li et al., 2024 (12)	China	32 healthy, 60 gingivitis (18–25)	Postbiotic (*L. paracasei* Probio-01, NR)	Blank toothpaste	Twice daily brushing for 3 months	BOP, GI, PI, Oral microbiota (plaque)	Significant reductions in BOP, GI, and PI; increased beneficial core bacteria such as Leptotrichia and Fusobacterium.
2	Rui et al., 2024 (13)	China	31 healthy (18–30)	Postbiotic (*L. salivarius LS97, L. paracasei LC86, L. acidophilus LA85*, 3 × 10^10^ CFU)	Blank toothpaste	Twice daily brushing for 2 months with follow-up 1 month after discontinuation	Salivary IgA levels, SCFAs levels, Oral microbiota (salivary)	Increased salivary IgA and beneficial acids; improved microbiota diversity; reduced harmful bacteria.
3	Lee et al., 2024 (14)	Taiwan	17 gingivitis (20–59)	Postbiotic (*L. paracasei GMNL-143*, 0.7 × 10^9^ cells/g)	Placebo toothpaste	Twice daily brushing for 4 weeks	GI, PI, Oral microbiota (plaque), GCF, Salivary IgA levels	Significant decrease in gingival index and reduction in *S. mutans* levels in GCF samples.
4	Butera et al., 2022 (15)	Italy	40 periodontitis (18–70)	Paraprobiotic (Lactobacillus, Bifidobacterium strains, NR)	0.2% Chlorhexidine-based toothpaste	Twice daily brushing for 6 months	PPD, PI, CAL, BOP, BS, SBI, API, AG, GR, Pathological Sites	Significant reduction of clinical indices and red complex.
5	Butera et al., 2021 (16)	Italy	60 periodontitis (18–70)	Probiotic (Lactobacillus, Bifidobacterium strains, NR)	0.2% Chlorhexidine-based toothpaste	Twice daily brushing for 6 months	PPD, PI, CAL, BOP, BS, SBI, API, AG, GR, Pathological Sites	Significant reduction of clinical indices and orange complex.
6	Selvaraj et al., 2020 (17)	India	60 healthy (18–30)	Probiotic (*L. paracasei*, NR)	Neem-based toothpaste	Twice daily brushing with toothpaste for 60 days	Levels of *S. mutans* were evaluated by CRT bacteria test	Effective reduction in *S. mutans* counts comparable to neem-based toothpaste.
7	Ballini et al., 2019 (18)	Italy	24 healthy (18–40)	Probiotic (*L. paracasei*, NR)	Non-active toothpaste	Twice daily brushing for 90 days	OHI, SI, *S. mutans* levels in saliva, remineralization	Reduction in OHI, SI, *S. mutans,* and enamel remineralization.
8	Prabakar et al., 2018 (19)	India	52 healthy (18–25)	Probiotic (NR, NR)	Green tea dentifrice, Fluoridated dentifrice, CHX dentifrice	Twice daily brushing with toothpaste for 30 days	Levels of *S. mutans* and Lactobacillus in plaque and saliva	Reduction in *S. mutans* and *Lactobacillus* counts.
9	Maden et al., 2018 (20)	Turkey	60 healthy (13–15)	Probiotic (*L. paracasei*, NR)	Fluoride toothpaste, Xylitol toothpaste	Twice daily brushing with toothpaste for 6 weeks	Levels of *S. mutans* and *Lactobacillus* were evaluated using CRT bacteria test	PerioBiotic probiotic toothpaste and fluoride toothpaste showed significant reduction in *S. mutans* and Lactobacillus; xylitol toothpaste showed no significant effect.
10	Alkaya et al., 2017 (21)	Turkey	40 gingivitis (18–31)	Probiotic (B. subtilis, B. megaterium, B. pumulus, 5 × 10^7^ CFU)	Placebo products	Twice daily brushing with probiotic products for 8 weeks	PI, GI, PPD, BOP, tongue coating	No significant differences between placebo and bacilli-containing products on gingivitis parameters.
11	Maden et al., 2017 (22)	Turkey	48 healthy (13–15)	Probiotic (*L. paracasei*, NR)	Fluoride toothpaste, Xylitol toothpaste	Twice daily brushing with toothpaste for 6 weeks	PI, GI	PerioBiotic showed superior effects in reducing both plaque and gingival indices; fluoride toothpaste was effective for both indices; xylitol alone showed limited gingival benefits.
12	Rubido et al., 2014 (23)	Spain	20 healthy (Not specified)	Probiotic (*S. salivarius, L. salivarius, B. bifidum, E. faecium, L. acidophilus, L. plantarum*, NR)	Sterile water	Once brushing with toothpaste for 1 day	Bacterial vitality in saliva, PI	Significantly increased plaque regrowth compared to water.

NR, not reported; AG, adherent gingiva; GBI, gingival bleeding index; PI, plaque index; BOP, bleeding on probing; GI, gingival index; GCF, gingival crevicular fluid; PPD, probing pocket depth; CAL, clinical attachment level; SI, stain index; CRT, caries risk test; API, approximal plaque index; BS, bleeding score; CHX, chlorhexidine; GR, gingival recession; IgA, immunoglobulin A; SBI, sulcus bleeding index; SCFAs, short-chain fatty acids; *L. paracasei*, *Lactobacillus paracasei*; red complex: *Porphyromonas gingivalis*, *Porphyromonas endodontalis, Tannerella forsythia*, *Troponema denticola, Peptostreptococcus micros, Filifactor alocis, Synergistetes*, *Aggregatibacter actinomycetemcomitans*; orange complex: *Prevotella intermedia*, *Fusobacterium nucleatum, Campylobacter rectus, Rothia dentocariosa, Leptotrichia hofstadii*.

### 3.3 Risk of bias

Overall, the risk of bias was identified as high in one study, with some concerns in seven studies, and low in four studies. As shown in Figure 2, the randomization process (D1) raised some concerns in six studies, primarily due to a lack of information on allocation sequence concealment. Deviations from intended interventions (D2) showed some concerns in four studies and unclear information in one study, while the remaining studies maintained low risk. Missing outcome data (D3) demonstrated high risk in one study (13) due to high dropout rates, whereas most other studies showed low risk. Measurement of outcomes (D4) was generally well controlled across all studies, with only low risk assessments. Selection of reported results (D5) raised some concerns in six studies, while the remaining studies showed low risk. The overall risk profile indicates that most studies maintained acceptable methodological quality, with only one study showing high overall risk.

### 3.4 Primary outcomes

The included studies investigated the use of various LAB-based toothpaste formulations for oral health. The probiotic strains in these toothpaste samples were predominantly *Lactobacillus* species, particularly *L. paracasei* (12–14, 17, 18, 20, 22). Other probiotic strains included Bifidobacterium (15, 16, 21, 23) and combination formulations (13, 15, 16, 21, 23). Treatment durations ranged from 1 day to 6 months, with most studies implementing a twice-daily protocol.

#### 3.4.1 PI

Oral hygiene was assessed in 8 studies (12, 14–16, 18, 21–23) with the PI, Turesky Modified Quigley-Hein Index, and Silness-Löe Index evaluated parameters. Most studies investigating LAB-based toothpaste reported significant improvements in plaque compared to the control groups. The magnitude of improvement varied across studies, likely reflecting differences in probiotic strains, concentrations, and treatment durations, with *L. paracasei* showing particularly consistent results.

#### 3.4.2 Periodontal parameters

Periodontal health parameters were evaluated in six studies (12, 14–16, 21, 22) by assessing the GI, BOP, probing pocket depth (PPD), and CAL. Evidence consistently demonstrated improvements in the gingival inflammation markers following probiotic interventions across various treatment durations ranging from 4 weeks to 6 months. For patients with established periodontitis (15, 16), significant improvements in the CAL and PPD were observed with 6-month treatment durations. Studies involving participants with gingivitis (12, 14, 21) showed notable reductions in the GI scores, with *L. paracasei* demonstrating consistent clinical improvements in the gingival inflammation markers.

#### 3.4.3 Other parameters

Additional parameters were assessed in two studies, including salivary immunoglobulin A (IgA) levels (13, 14) and short-chain fatty acid (SCFA) levels (13). Rui et al. (13) evaluated both salivary IgA and SCFAs following postbiotic toothpaste application, reporting increased salivary IgA and beneficial acids after 2 months of use, with continued benefits observed 1 month after discontinuation. Lee et al. (14) assessed salivary IgA levels in participants with gingivitis, showing a significant decrease in the salivary IgA levels in one experimental series after 4 weeks of *L. paracasei* GMNL-143 toothpaste use; however, no statistically significant difference was detected in the combined analysis.

### 3.5 Secondary outcomes

Microbiological parameters were examined in seven studies (12–14, 17–20). *Streptococcus mutans* levels were the most frequently evaluated parameter (four studies) (17–20), followed by *Lactobacillus* counts (two studies) (19, 20) and oral microbiota composition analyses (three studies) (12–14). All four studies measuring the *S. mutans* levels reported significant reductions (17–20), while both studies evaluating *Lactobacillus* counts demonstrated decreased levels (19, 20). Studies investigating oral microbiota composition reported increased beneficial bacteria and improved microbial diversity (12–14).

### 3.6 Meta-analysis

Of the 12 included studies, five provided data suitable for quantitative synthesis. Raw data were requested from additional study authors to facilitate meta-analysis; however, these requests were not fulfilled. To ensure clinical homogeneity and meaningful statistical synthesis, meta-analysis was restricted to four studies involving participants with periodontal pathology (gingivitis and periodontitis).

#### 3.6.1 Pi

**Figure 3:**
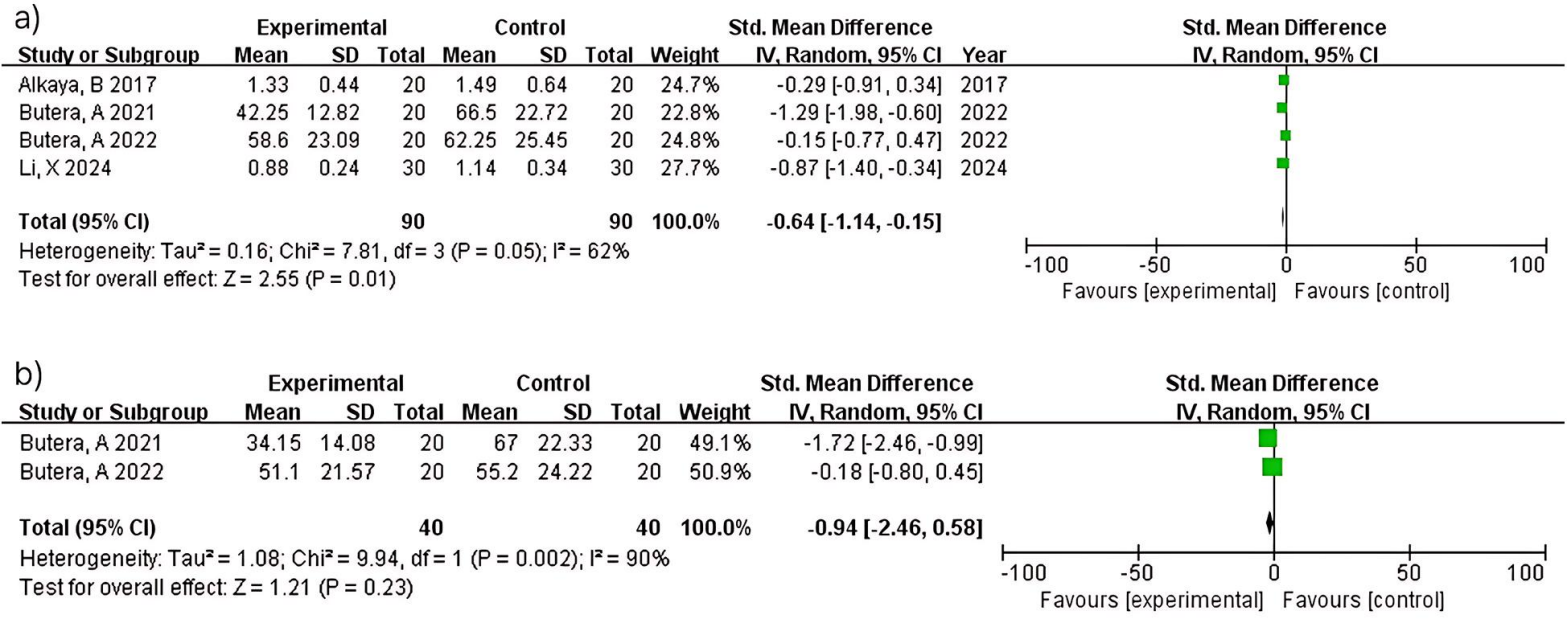
Plaque Index (PI) at ≥3 **(a)** and 6 months **(b)**.

Meta-analysis of four studies (*n* = 180) demonstrated significant plaque reduction with LAB-based toothpaste [MD = −0.64; 95% confidence interval (CI): −1.14 to −0.15; *p* = 0.01]. Substantial heterogeneity was observed (I^2^ = 62%), likely reflecting differences in probiotic strains (L. paracasei Probio-01 vs. GMNL-143), concentrations (not reported vs. 0.7 × 10^9^ cells/g), and study populations between studies (12) and (14). At 6 months, two studies (*n* = 80) showed non-significant plaque reduction (MD = −0.94; 95% CI: −2.46–0.58; *p* = 0.23) with considerable heterogeneity (I^2^ = 90%). Despite both studies (15, 16) using similar methodologies, heterogeneity may reflect differences in probiotic formulations and baseline periodontal severity. The pooled estimate should be interpreted with extreme caution (Figure 3).

#### 3.6.2 Periodontal parameters

##### 3.6.2.1 Gi

**Figure 4:**
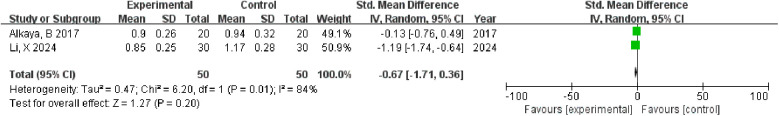
Gingival Index (GI) (within 2 months).

Two studies (*n* = 100) showed non-significant gingival improvement (MD = −0.67; 95% CI: −1.71–0.36; *p* = 0.20) with considerable heterogeneity (I^2^ = 84%). Heterogeneity reflects differences in the probiotic strains, concentrations, intervention duration (3 months vs. 4 weeks), and study populations between studies (12) and (14) (Figure 4).

##### 3.6.2.2 PPD

**Figure 5:**
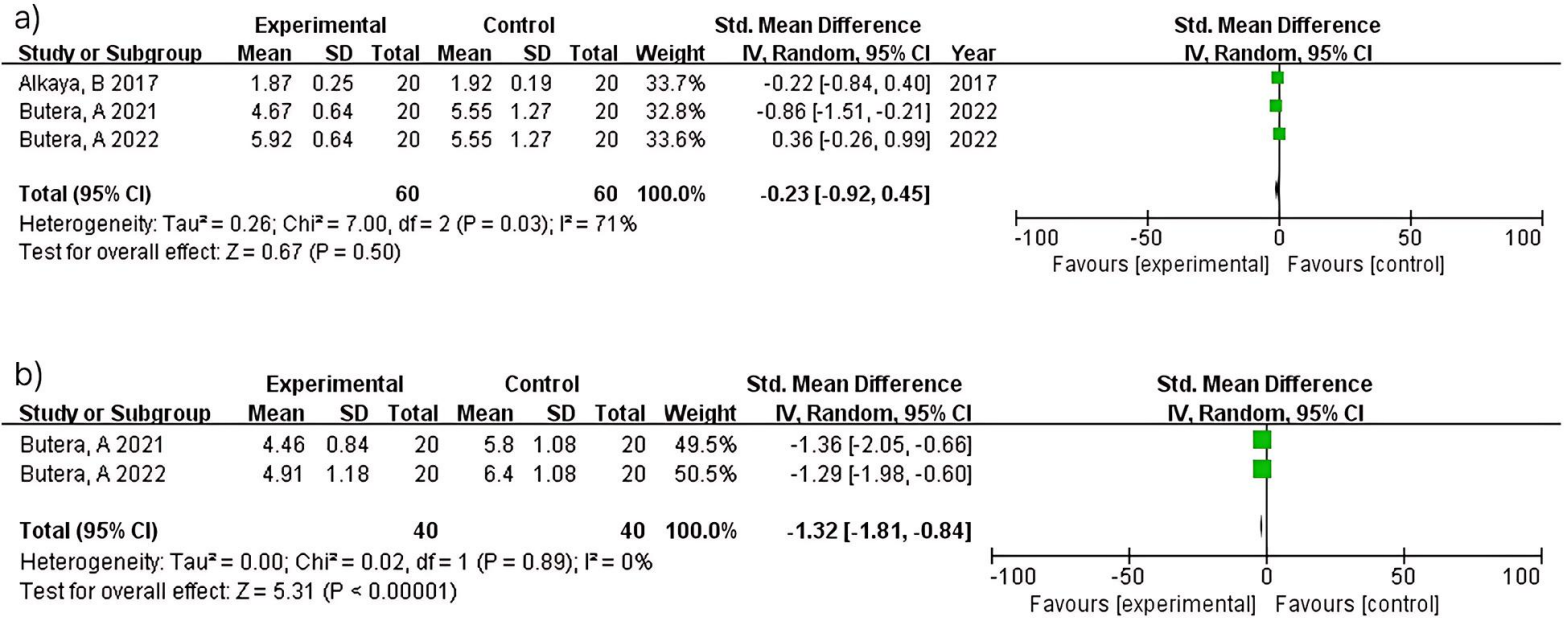
Probing pocket depth (PPD) at ≥3 **(a)** and 6 months **(b)**.

Three studies (*n* = 120) within 3 months showed no significant difference (MD = −0.23; 95% CI: −0.92–0.45; *p* = 0.50) with substantial heterogeneity (I^2^ = 71%). Heterogeneity reflects differences in probiotic strains (*L. paracasei* vs. Bacillus species), intervention duration, and study populations across studies (12, 14, 21). In contrast, two studies (*n* = 80) at 6 months demonstrated significant probing depth reduction (MD = −1.32; 95% CI: −1.81 to −0.84; *p* < 0.00001) with no heterogeneity (I^2^ = 0%). The consistent findings between studies (15, 16) reflect standardized protocols in periodontitis patients using similar probiotic formulations over 6 months (Figure 5).

##### 3.6.2.3 BOP

**Figure 6:**
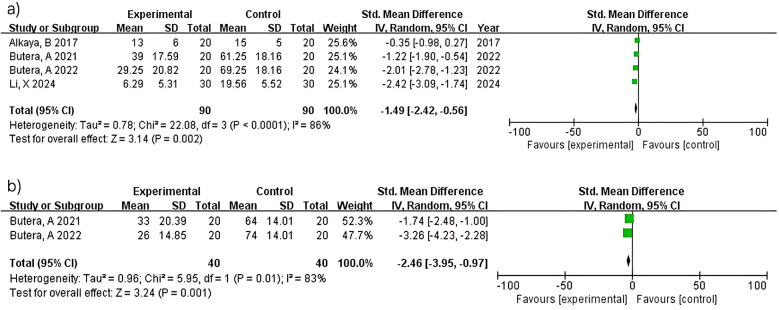
Bleeding on probing (BOP) at ≥3 **(a)** and 6 months **(b)**.

Four studies (*n* = 180) within 3 months showed significant bleeding reduction (MD = −1.49; 95% CI: −2.42 to −0.56; *p* = 0.002) with considerable heterogeneity (I^2^ = 86%). Heterogeneity reflects diversity in probiotic strains (*L. paracasei* variants vs. Bacillus species), intervention duration, and study populations across multiple research groups. At 6 months, two studies (*n* = 80) demonstrated greater bleeding reduction (MD = −2.46; 95% CI: −3.95 to −0.97; *p* = 0.001) with considerable heterogeneity (I^2^ = 83%). Despite both studies (15, 16) using similar methodologies, heterogeneity may reflect differences baseline bleeding severity (Figure 6).

##### 3.6.2.4 CAL

**Figure 7:**
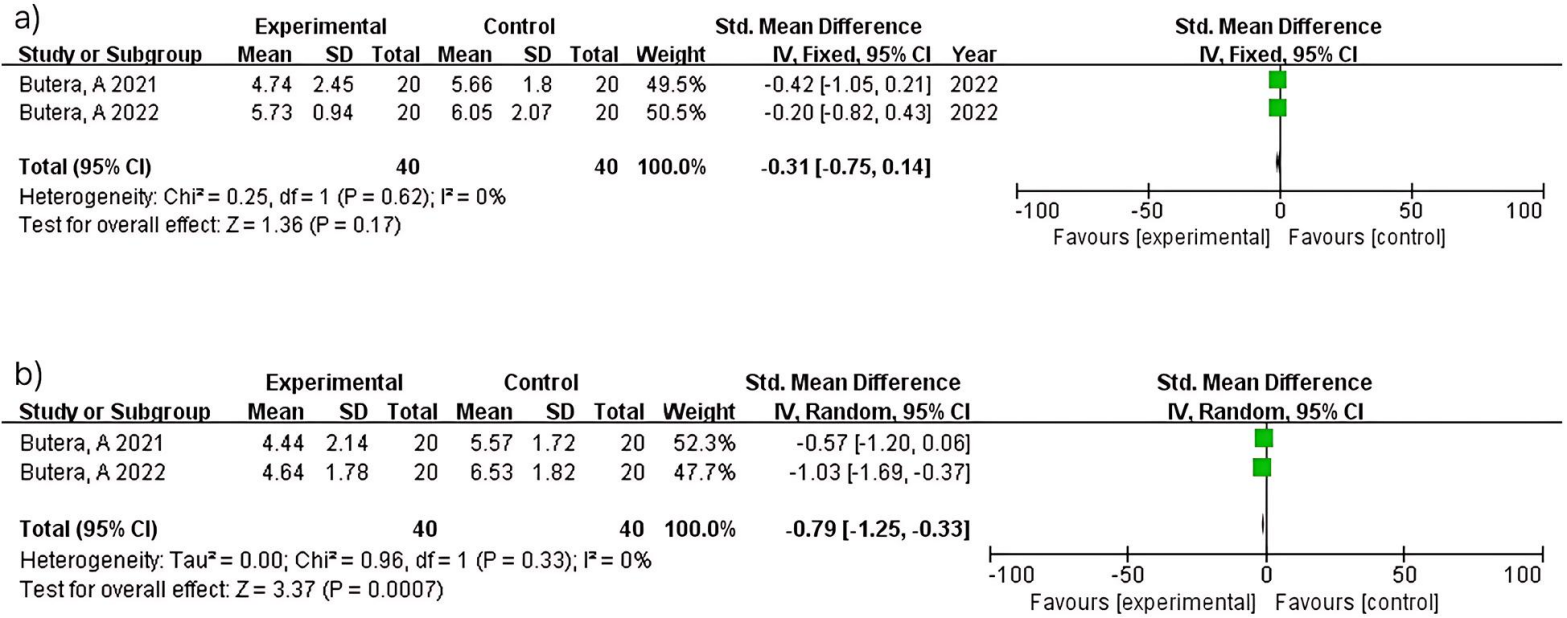
Clinical attachment level (CAL) at 3 **(a)** and 6 months **(b)**.

Two studies (*n* = 80) within 3 months showed no significant difference (MD = −0.31; 95% CI: −0.75–0.14; *p* = 0.17) with no heterogeneity (I^2^ = 0%). Consistent results between studies (15, 16) reflect standardized protocols in periodontitis patients. In contrast, two studies (*n* = 80) at 6 months demonstrated significant attachment level improvement (MD = −0.79; 95% CI: −1.25 to −0.33; *p* = 0.0007) with no heterogeneity (I^2^ = 0%). Consistent findings between studies (15, 16) support the reliability of longer intervention periods for periodontal healing (Figure 7).

#### 3.6.3 Total bacterial count

**Figure 8:**
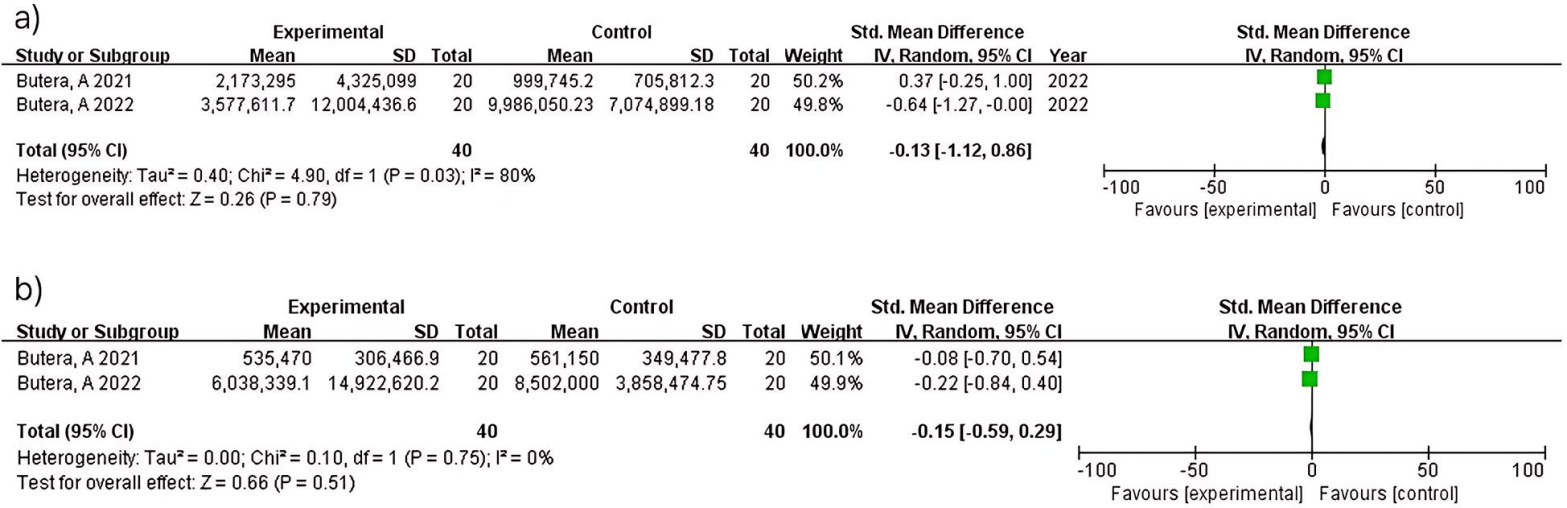
Total bacteria count at 3 **(a)** and 6 months **(b)**.

Two studies (*n* = 80) at 3 months showed no significant difference (MD = −0.13; 95% CI: −1.12–0.86; *p* = 0.79) with considerable heterogeneity (I^2^ = 80%). At 6 months, the same two studies (*n* = 80) demonstrated no significant difference (MD = −0.15; 95% CI: −0.59–0.29; *p* = 0.51) with no heterogeneity (I^2^ = 0%). The heterogeneity observed at 3 months contrasts with the homogeneity at 6 months between studies (15, 16), though interpretation is limited by the small number of included studies (Figure 8).

### 3.7 Summary of findings

We assessed the certainty of evidence using the GRADE approach, with results presented in the Summary of Findings table (Table 2). The certainty of evidence ranged from very low to moderate across outcomes, with substantial heterogeneity as the primary limiting factor. Both short-term outcomes (plaque index and bleeding on probing at 3 months) were downgraded for risk of bias and inconsistency. Plaque index showed low certainty with I^2^ = 62%, while bleeding on probing showed very low certainty due to more serious inconsistency (I^2^ = 86%). The evidence for both probing pocket depth and clinical attachment level at 6 months showed moderate certainty, downgraded for imprecision due to small sample size (*n* = 80 from 2 studies) despite no heterogeneity (I^2^ = 0%). Assessment of publication bias was not feasible due to insufficient studies per outcome (<10 studies).

**Table 2:** Summary of findings for the main comparison.

Lactic acid bacteria-based toothpaste compared to control for oral health			
Patients or population: Adults and adolescents with healthy oral condition, gingivitis, or periodontitis Settings: Community-based dental Intervention: Lactic acid bacteria-based toothpaste (containing probiotics, prebiotics, synbiotics, or postbiotics) Comparison: Placebo toothpaste, conventional toothpaste, or no treatment			
Outcomes	Impact	Number of participants (Studies)	Certainty of the evidence (GRADE)
PI (Follow-up: 3 months)	MD 0.64 lower (95% CI:-1.14 to −0.15)	180 (4 RCTs)	_⊕⊕⊖⊖_ Low^a^^,^^b^
BOP (Follow-up: 3 months)	MD 1.49% lower (95% CI: −2.42 to −0.56)	180 (4 RCTs)	_⊕⊖⊖⊖_ Very low^a^^,^^c^
PPD (Follow-up: 6 months)	MD 1.32 mm lower (95% CI: −1.81 to −0.84)	80 (2 RCTs)	_⊕⊕⊕⊖_ Moderate^c^
Clinical Attachment Level (CAL) (Follow-up: 6 months)	MD 0.79 mm improvement (95% CI: −1.25 to −0.33)	80 (2 RCTs)	_⊕⊕⊕⊖_ Moderate^c^
PI, plaque index; BOP, bleeding on probing; PPD, probing pocket depth; CAL, clinical attachment level; MD, mean difference; RCT, randomized controlled trial * GRADE Working Group grades of evidence **High** = This research provides a very good indication of the likely effect. The likelihood that the effect will be substantially different is low. **Moderate** = This research provides a good indication of the likely effect. The likelihood that the effect will be substantially different is moderate. **Low** = This research provides some indication of the likely effect. However, the likelihood that it will be substantially different is high. **Very low** = This research does not provide a reliable indication of the likely effect. The likelihood that the effect will be substantially different is very high.			

a Limitations of study designs.

b Inconsistency of results.

c Imprecision of results.

### 3.8 Narrative subgroup analysis

#### 3.8.1 Population-based analysis

Given the substantial clinical heterogeneity observed in study populations and the small number of studies suitable for meta-analysis, we conducted a narrative subgroup analysis based on participant oral health status to better understand the differential effects of LAB-based toothpaste across different populations. Healthy individuals (7 studies, *n* = 257) showed beneficial effects primarily involving plaque control and bacterial reduction (13, 17–20, 22). Studies using *L. paracasei* strains consistently reported positive outcomes (17–20, 22), while one study using multiple probiotic strains showed increased plaque regrowth (23). Patients with gingivitis (3 studies, *n* = 149) demonstrated variable responses depending on probiotic strain selection. Studies using *L. paracasei* formulations showed beneficial effects on gingival inflammation parameters (12, 14), while Bacillus species showed no significant differences compared to placebo (21). Patients with periodontitis (2 studies, *n* = 100) showed consistent beneficial effects on clinical periodontal parameters when treated with *Lactobacillus* and Bifidobacterium formulations for 6 months (15, 16). Both studies reported significant improvements in periodontal indices and reductions in pathogenic bacterial complexes. These findings suggest that treatment efficacy depends on both target population and specific probiotic strain selection, with *L. paracasei* showing consistent benefits across healthy and gingivitis populations (12, 14, 17–20, 22), while *Lactobacillus* and Bifidobacterium combinations showed effectiveness in patients with periodontitis (15, 16). However, some formulations may result in increased plaque regrowth (23), emphasizing the importance of appropriate strain selection.

#### 3.8.2 Strain-based analysis

*L. paracasei* strains showed the most consistent efficacy across different populations and formulations (7/7 studies positive) (12, 14, 17, 18, 20, 22). Both postbiotic and live probiotic formulations demonstrated beneficial effects on plaque control, gingival inflammation, and bacterial reduction. *Lactobacillus* and Bifidobacterium combinations showed highly consistent results in patients with periodontitis (2/2 studies positive) (15, 16), particularly when used as long-term adjunctive therapy with standardized protocols. Bacillus species showed limited efficacy (21), with no significant benefits observed for gingivitis parameters compared to placebo. Mixed probiotic formulations showed variable results (2/3 studies positive) (13, 19, 23), suggesting that effectiveness may depend on specific strain combinations and intervention protocols. These findings emphasize the critical importance of strain selection in LAB-based oral care products, with *L. paracasei* demonstrating the most reliable efficacy across diverse clinical applications, while *Lactobacillus* and Bifidobacterium combinations showed specific effectiveness for periodontitis management.

## 4 Discussion

This systematic review and meta-analysis evaluated the efficacy of LAB-based toothpaste on various oral health parameters through analysis of 12 RCTs published between 2014 and 2024. While the overall direction of effects consistently favored LAB-based interventions across multiple oral health domains, the substantial heterogeneity observed in most meta-analyses (I^2^ > 75%) limits the precision of pooled estimates and requires cautious interpretation of results. This heterogeneity significantly impacted evidence certainty, as demonstrated in our GRADE Summary of Findings (Table 2), where most outcomes were downgraded for inconsistency and imprecision. Despite efforts to explore sources of heterogeneity through subgroup and sensitivity analyses, the considerable variation remained largely unexplained due to the limited number of studies available for each outcome and differences in probiotic strains, concentrations, and study populations across research groups.

Short-term interventions (≤3 months) demonstrated significant benefits for plaque control and bleeding reduction with substantial heterogeneity (I^2^ = 62%–86%), reflecting diversity in probiotic strains, concentrations, and study populations. The heterogeneity observed between studies (12, 14) for plaque and gingival indices can be attributed to differences in probiotic strains (*L. paracasei* Probio-01 vs. GMNL-143), concentrations (not reported vs. 0.7 × 10^9^ cells/g), and intervention durations (3 months vs. 4 weeks). Additionally, studies using different probiotic species, such as Bacillus strains in study (21), showed markedly different response patterns compared to *L. paracasei* formulations.

Longer interventions (≥6 months) demonstrated remarkably consistent effects for periodontal parameters (I^2^ = 0%) when standardized protocols were used in similar populations (5, 16), suggesting that sustained use may be necessary for predictable periodontal benefits. This perfect consistency between studies by the same research group using standardized methodologies provides the most reliable evidence from this meta-analysis, indicating that treatment duration and protocol standardization are critical factors for achieving consistent clinical outcomes. The heterogeneity patterns indicate that *L. paracasei* strains show more consistent effects than Bacillus species, and that probiotic strain selection, dosage, and treatment duration critically influence outcomes. Studies using standardized protocols (15, 16) showed perfect consistency for periodontal parameters, while studies using different formulations or populations showed high variability, emphasizing the importance of treatment protocol standardization in clinical practice.

The meta-analysis demonstrated statistically significant improvements in the PI and BOP; however, the wide prediction intervals suggest that the magnitude of benefit may vary considerably across different clinical settings. This aligns with the growing body of evidence supporting the role of probiotics in modulating biofilm formation and composition (24). Observed plaque reductions were consistent across studies using *L. paracasei* strains, with a study (18) reporting up to a 76% reduction in the oral hygiene index over 90 days, suggesting that probiotic interventions could provide meaningful clinical benefits for plaque control.

*Lactobacillus* species, particularly *L. paracasei*, have been shown to inhibit bacterial adhesion to oral surfaces by competing for binding sites and producing biosurfactants (25). Additionally, many LAB produce bacteriocins and hydrogen peroxide, which directly inhibit the growth of cariogenic and periodontopathogenic bacteria (26). The consistent clinical efficacy of *L. paracasei* across studies may be attributed to strain-specific characteristics including enhanced acid tolerance (27), superior oral colonization capacity through adhesion and aggregation properties (25), and favorable immunomodulatory effects in periodontal therapy (28), contributing to more predictable clinical outcomes across diverse patient populations. The variability in plaque reduction efficacy across studies likely reflects differences in participant characteristics, probiotic strains used, and treatment durations.

LAB-based toothpaste with long intervention periods significantly improved key periodontal parameters, particularly PPD and CAL. The significant improvements observed in PPD and CAL suggest that probiotics exert an immunomodulatory effect (6). LAB strains modulate immune responses by reducing pro-inflammatory cytokines (IL-1β, TNF-α) while increasing anti-inflammatory cytokines (IL-10), creating conditions that limit tissue destruction and promote periodontal healing (28, 29) This immunomodulatory effect reduces matrix metalloproteinase activity, particularly MMP-8, while enhancing tissue inhibitor of metalloproteinase-1 (TIMP-1) expression, thereby limiting periodontal tissue degradation (30). The resulting anti-inflammatory environment promotes tissue repair processes that facilitate new attachment formation and CAL gain, with PPD reduction occurring through inflammatory edema resolution and enhanced junctional epithelium integrity (31, 32).

Recent research has demonstrated that specific probiotic strains can reduce levels of pro-inflammatory cytokines [e.g., interleukin (IL)-1β and IL-8] and increase levels of anti-inflammatory cytokines, such as IL-10 in periodontal tissues (33). The reductions in gingival inflammation markers likely reflect the production of anti-inflammatory mediators and inhibition of pro-inflammatory cytokines by probiotics. For periodontal parameters, such as PPD and CAL improvements were observed only with longer intervention durations (≥6 months), and only when heterogeneity was minimal (I^2^ = 0%), suggesting more consistent effects for these outcomes under specific conditions. Our findings align with previous research demonstrating that measurable improvements in the pocket depth and CAL may require longer intervention periods with probiotic supplementation (31). The significant improvements observed in the PI and periodontal parameters are consistent with previous research showing that probiotics may disrupt biofilm formation through competitive exclusion of pathogenic bacteria and production of antimicrobial substances (5, 34). While gingival index showed improvement trends, the variable effects across different inflammatory markers suggest that probiotic mechanisms may affect different aspects of periodontal health with varying degrees of efficacy.

Our meta-analysis demonstrated no statistically significant differences in the total bacterial count between probiotic and control groups, with considerable heterogeneity despite methodological consistency between studies (15, 16). This finding is consistent with variable microbiological results observed across individual studies. While some studies reported significant reductions in periodontopathogens (19, 20), others found lower levels of pathogenic bacteria without reaching statistical significance (13, 15, 23).

The results regarding microbial diversity and composition were inconsistent. Some studies found no significant alterations in plaque microbiota diversity (14), while others observed enhanced microbial alpha diversity and reduced harmful bacteria without significantly affecting overall salivary microbiota structure (13). This heterogeneity may be attributed to variations in the probiotic strains, assessment methodologies, and target populations. The findings suggest that LAB-based toothpaste may exert beneficial effects through qualitative changes in microbial composition rather than quantitative reductions in the total bacteria.

From a clinical perspective, while the meta-analysis provides statistical evidence of benefit, the substantial heterogeneity observed suggests that the effectiveness of LAB-based toothpaste may vary considerably depending on patient characteristics, specific probiotic strains used, and treatment protocols. LAB-based toothpaste effectiveness depends heavily on specific probiotic strains, concentrations, and patient populations. *L. paracasei* strains showed consistent benefits across different studies, while Bacillus species showed mixed results, with a study (21) showing no significant differences between placebo and bacilli-containing products on the gingival parameters. The prediction intervals calculated for several outcomes indicate that in some clinical contexts, the intervention may provide substantial benefits, while in others, effects may be minimal or absent. This variability emphasizes the importance of considering individual patient factors and using evidence-based selection criteria when recommending LAB-based oral care products. Healthcare providers should consider that different LAB formulations may not be clinically equivalent, and the choice of specific probiotic strains, concentrations, and treatment duration appears to be critical for achieving optimal outcomes.

Probiotics also have potential systemic benefits in specific populations, such as improved glycemic control in patients with periodontal disease (35). This indicates that oral probiotic applications may have effects beyond the local oral environment, potentially influencing systemic inflammatory processes. Sustained use (≥6 months) with standardized protocols appears necessary for predictable periodontal benefits in periodontitis patients.

From a clinical perspective, these findings suggest that LAB-based toothpastes may serve as valuable adjunctive tools in routine oral care, particularly for patients with gingivitis or mild periodontal inflammation. The observed improvements in plaque control and gingival bleeding within 3 months indicate potential clinical benefits when used alongside conventional oral hygiene measures. However, clinicians should note that *Lactobacillus paracasei* formulations demonstrated the most consistent efficacy, and sustained use for at least 6 months appears necessary for periodontal benefits in patients with established periodontitis. Given the substantial heterogeneity in treatment responses, individual patient monitoring and strain-specific product selection will be important for optimizing clinical outcomes.

This review has several important limitations. First, the substantial heterogeneity observed in most meta-analyses (I^2^ > 75%) significantly limits the generalizability of pooled estimates, reflecting diversity in study populations, intervention protocols, and outcome measures. This heterogeneity was the primary factor limiting evidence certainty in our GRADE assessment. Second, the small number of studies suitable for quantitative synthesis (2–5 studies per outcome) results in imprecise estimates with wide confidence intervals. Assessment of publication bias using funnel plots was not feasible due to the limited number of studies suitable for meta-analysis. Cochrane guidelines recommend a minimum of 10 studies for meaningful interpretation of funnel plot asymmetry, limiting our ability to detect potential publication bias. Third, the overall risk of bias assessment revealed high risk in one study and some concerns in seven studies, potentially affecting reliability of findings. Fourth, many included studies used formulations containing multiple probiotic components (prebiotics, postbiotics, various probiotic strains), making it impossible to isolate the specific contribution of individual LAB strains to observed clinical effects. This confounding limits our ability to attribute benefits to specific probiotic mechanisms and affects the precision of effect estimates for individual LAB interventions. Fifth, unavailability of raw data limited our meta-analysis to a subset of included studies. Sixth, despite applying no language restrictions in our search strategy, all included studies were published in English, which may indicate language bias and the potential omission of relevant non-English publications.

Despite these limitations, this systematic review provides comprehensive evidence that probiotics can effectively reduce gingival inflammatory markers and plaque accumulation, though the moderate to very low certainty of evidence requires cautious clinical interpretation.

Future studies should prioritize: (i) standardization of intervention protocols including specific strains, concentrations, and treatment durations; (ii) consistent outcome measures and assessment timing; (iii) identification of patient subgroups most likely to benefit; and (iv) large-scale RCTs with adequate power and longer follow-up periods. Short-term interventions dominate current research; long-term efficacy data and safety profiles, including potential microbial resistance, are lacking and represent important areas for future investigation.

## 5 Conclusions

LAB-based toothpaste demonstrates beneficial effects on plaque control and gingival inflammation across diverse populations. However, substantial heterogeneity limits effect estimate precision, indicating that benefits may depend on specific patient characteristics, probiotic strains, and treatment protocols. *L. paracasei* strains showed consistent benefits, while sustained use (≥6 months) appears necessary for periodontal improvements. Standardized research protocols and larger, more homogeneous studies are warranted to provide reliable evidence for clinical practice recommendations.

## Data Availability

The original contributions presented in the study are included in the article/Supplementary Material, further inquiries can be directed to the corresponding author.
